# Reducing Loneliness in Stationary Geriatric Care with Robots and Virtual Encounters—A Contribution to the COVID-19 Pandemic

**DOI:** 10.3390/ijerph18094846

**Published:** 2021-05-01

**Authors:** Andreas Follmann, Franziska Schollemann, Andrea Arnolds, Pauline Weismann, Thea Laurentius, Rolf Rossaint, Michael Czaplik

**Affiliations:** 1 Department of Anesthesiology, University Hospital RWTH Aachen, Pauwelsstrasse 30, 52074 Aachen, Germany; fschollemann@ukaachen.de (F.S.); andrea.arnolds@gmx.de (A.A.); paweismann@ukaachen.de (P.W.); rrossaint@ukaachen.de (R.R.); mczaplik@ukaachen.de (M.C.); 2 Docs in Clouds TeleCare GmbH, Vaalser Straße 460, 52074 Aachen, Germany; 3 Department of Geriatric Medicine, University Hospital RWTH Aachen, Pauwelsstrasse 30, 52074 Aachen, Germany; tlaurentius@ukaachen.de

**Keywords:** video telephony, robotics, loneliness, social isolation, COVID-19, bans on visitors

## Abstract

The bans on visiting nursing homes during the COVID-19 pandemic, while intended to protect residents, also have the risk of increasing the loneliness and social isolation that already existed among the older generations before the pandemic. To combat loneliness and social isolation in nursing homes, this trial presents a study during which social networks of nursing home residents and elderly hospital patients were maintained through virtual encounters and robots, respectively. The observational trial included volunteers who were either residents of nursing homes or patients in a geriatric hospital. Each volunteer was asked to fill in a questionnaire containing three questions to measure loneliness. The questionnaire also documented whether video telephony via the robot, an alternative contact option (for example, a phone call), or no contact with relatives had taken place. The aim was to work out the general acceptance and the benefits of virtual encounters using robots for different roles (users, relatives, nursing staff, facilities). Seventy volunteers with three possible interventions (non-contact, virtual encounters by means of a robot, and any other contact) took part in this trial. The frequency of use of the robot increased steadily over the course of the study, and it was regularly used in all facilities during the weeks of visitor bans (*n* = 134 times). In the hospital, loneliness decreased significantly among patients for whom the robot was used to provide contact (F(1,25) = 7.783, *p* = 0.01). In the nursing homes, no demonstrable effect could be achieved in this way, although the subject feedback from the users was consistently positive.

## 1. Introduction

Even before the COVID-19 pandemic, a nonsignificant proportion of the global elderly population felt lonely, depending on the country [1]. In a study in US, 29% of senior citizens surveyed said they felt lonely [2]. This trend may be worsened by the COVID-19 pandemic due to social distancing and bans on visitors to protect residents from infection with the severe acute respiratory syndrome-coronavirus 2 (SARS-CoV-2) virus in fully stationary nursing homes. The residents of these institutions are also high-risk groups, as 29–72% of COVID-19 deaths in Europe have been among residents of full inpatient facilities [3], more than 95% of the deaths caused by COVID-19 occurred in senior citizens older than 60 [4]. In 2020, the COVID-19 pandemic led to a ban on visits to nursing homes as one of several precautionary measures [5]. This radical procedure is important for senior citizens because not only do they define a high-risk group but also the virus happens to be highly contagious in close person-to-person contact through coughing or sneezing respiratory droplets or aerosols [6]. Moreover, the virus may remain infectious in the air or on surfaces up to 24 h depending on the material and environment [7]. With no vaccination available at the beginning of the COVID-19 pandemic, interventions such as quarantining infected individuals and their family members, contact tracing, and social distancing were recommended based on modeling studies [8,9]. Some researchers have suggested extending the interventions, including social distancing, for more years because there is a possibility that this virus will turn into seasonal influenza after the pandemic [10]. Thus, careful considerations are required for the people who may suffer from the risk of infection and the possible intervention extensions.

Maintaining healthy lifestyles and mental health in isolation is comparatively difficult for residents of fully inpatient care facilities, especially with the additional stress factor of visitor bans. Added to this are the physical limitations that make seemingly everyday things such as climbing stairs and carrying shopping more difficult for elderly people with increasing age [11]. At the same time, the aspect of physical limitations does not affect both genders equally, gender seems to correlate with limitations differently than the body mass index [12]. Therefore, the senior citizens are often dependent on the support from communities to maintain daily routines and stay active. It was shown that a longer quarantine duration acts as a stressor for people, as do the boredom, inadequate supplies, and inadequate information during the quarantine. These stressors showed negative psychological effects, such as post-traumatic stress symptoms, confusion, and anger [13]. In addition, social isolation and loneliness were associated as higher risk factors for progression of frailty [14] and mortality [15].

Questionnaires serve as a tool in the sociophysiological environment to measure loneliness and social isolation. For example, the Revised UCLA (University of California, Los Angeles) Loneliness scale is based on 20 items, with 10 items dealing with satisfaction reflection and the other 10 dealing with dissatisfaction regarding social relationships [16]. The disadvantage of such an extensive questionnaire is that it can hardly be used in a long-term study. To simplify the measurement of loneliness, Hughes et al. [17] developed a three-item loneliness scale suited for large-scale surveys, which simultaneously measures the overall loneliness quite well. As an intervention to reduce loneliness and social isolation, video calls are recommended to maintain the social network [18,19]. Innovative solutions are especially needed in this area because of ongoing demographic changes. Within a scoping review, the analysis of 61 studies dealing with the use of robots in elderly care was divided into five groups: affective therapy, cognitive training, social facilitator, companionship, and physiological therapy [20]. This shows that—also regarding demographic change—innovative solutions are needed in this area. Residents of fully residential institutions often do not have opportunities to participate in studies, and thus this study reaches a scientifically interesting target group and can contribute to help the pandemic situation.

The home care robot temi (Medisana GmbH, Neuss, Germany) provides senior citizens easy access to a digital platform for communication with their families, caretakers, and friends. Temi is an autonomously driving humanoid robot that can be operated by voice control and is, therefore, easy to use even for people who are not familiar with digital devices. Other studies have suggested that a voice control assistant makes it easier for older people to use digital media [21]. For this reason, temi was suitable for this application. With the help of the robot, the residents of senior citizen facilities were able to perform video calls.

In this publication, our aim is to analyze whether the temi is used and if it is sufficiently accepted by the elderly, but also the nursing staff. Furthermore, it must be evaluated whether the temi is able to combat loneliness and social isolation. Therefore, we would like to evaluate the acceptance of video calls and their benefits to senior citizens using temi in fully stationary nursing homes and a hospital. As a secondary parameter, a questionnaire was used to ask about the loneliness of the residents.

## 2. Materials and Methods

### 2.1. Type of Study

Within this implementation study, data were acquired in three different facilities: two cooperating fully stationary nursing homes in rural areas (in the German districts of Heinsberg and Euskirchen), as well as in the department of elderly care (University Hospital RWTH Aachen, Aachen, Germany). The data collection took place in the form of questionnaires, which the participants filled out independently if possible. Thus, a total of 573 questionnaires could be acquired.

### 2.2. Sampling

The questionnaires of *n* = 70 participants were collected for the data evaluation. The participants were 83 years old on average and were majority female (19 men, 51 women).

### 2.3. Inclusion and Exclusion Criteria of the Participants

Informed consent was required for inclusion in the study. At the same time, the volunteers’ relatives also declared their willingness to be contacted. As another inclusion criteria for the virtual encounter group (using the temi robot), residents and patients were included who were assessed by the nursing staff as physically and mentally capable of dealing with technology. Depending on the nurses’ assessments, all other interested persons were assigned to the control group.

### 2.4. Procedure

**Figure 1:**
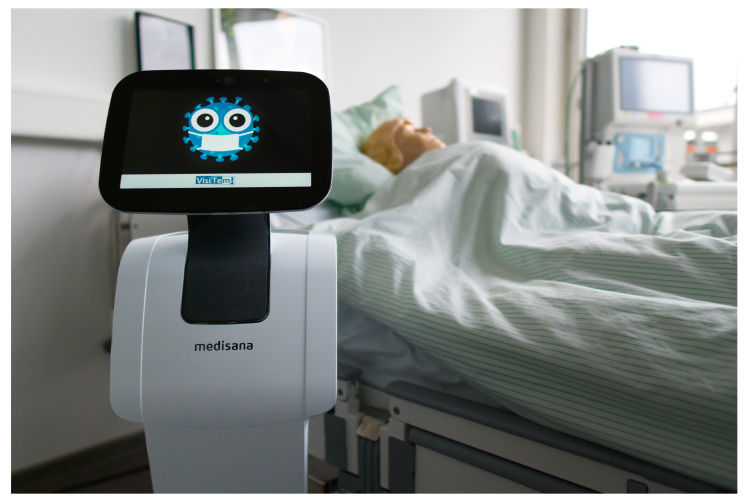
Within the study, the home care robot temi (Medisana GmbH, Neuss, Germany) was used to establish video telephony between relatives and residents of two elderly care facilities and patients of a geriatric clinic via the Skype application during COVID-19 pandemic visitor bans.

To initiate communication via video telephony and to rebuild the contact to relatives that was missing due to visitor bans, three temi robots (Figure 1) were used simultaneously in two nursing homes as well as a hospital. With the help of these robots, video telephony can be established between the patients or residents of the facilities and their relatives. For this purpose, *Skype* (Skype Technologies SA, Palo Alto, CA, USA) was used, and appointments between relatives and residents were coordinated. During the appointments, the residents’ relatives used temi to make video calls, during a one-hour window, with their relatives in the respective facilities.

### 2.5. Study Variables

During a two-month period of the pandemic in 2020, the virtual encounter group had the opportunity to meet their relatives virtually by means of temi. Intended as a quick solution to the problem of social isolation during the visitor bans, the use of temi was evaluated in terms of acceptance and feasibility.

Temi usage: Here, the use of the temi was analyzed in terms of the number of virtual encounters during the study period.

Loneliness score: Furthermore, a short, scientifically established questionnaire on loneliness and social isolation by Hughes et al. [17] was completed, ideally daily, as a further scientific parameter (Table 1).

The loneliness score can be calculated as the sum of the points of each of three questions. Therefore, values from 3 to 9 can be achieved. The higher the score, the greater the measured feeling of loneliness. The questionnaires were collected over the study period, regardless of whether video telephony using temi had been established. Alternative ways of contacting people outside the facilities were also documented in the questionnaires, such as telephone calls and "window visits," personal meetings through a window to have a barrier from aerosols and droplets. The type of intervention (virtual encounter using temi, non-contact, or alternative (calls, window visits or video call using a tablet)) was noted on the questionnaires by the nursing staff. The only questionnaires valid for the analysis defined the intervention that took place, a unique assignment to a volunteer, and completion of all three loneliness score questions.

Benefits of the temi use: As an additional variable, it is important to work out the benefits of using the robots for the different roles involved. In a short telephone interview at the end of the study, advantages for the respective user groups were worked out. The feedback given was summarized and assigned to the respective roles.

### 2.6. Ethical Aspects

The local ethics committee (EK 143/20) approved the study. The data was collected in a pseudonymized form, which was used to assign the questionnaires over time.

### 2.7. Statistical Analysis

IBM SPSS Statistics 24 (International Business Machines Corporation, Armonk, NY, USA) was used for statistical analysis. A single-factor analysis of variance was applied for the subgroup analysis. In addition, the effect of the treatment on the average loneliness score was investigated using ANOVA with repeated measurements. The significance level chosen for the entire analysis was *p* = 0.05. The loneliness scores were first analyzed over time, where, however, no significance could be detected. For this reason, the average loneliness score was analyzed as a function of the intervention.

## 3. Results

The study includes a survey of 70 volunteers of two nursing homes and one hospital. 19 men and 51 women took part in the survey, who were 83 years old on average (range 59 to 98 years). The total observation time was between 1 and 78 days – IQR (3;24). The participants were interviewed between 1 and 37 times – IQR (3;5). During the study, 623 questionnaires were acquired. Due to not meeting the necessary criteria for inclusion, 36 questionnaires were excluded. One volunteer declined to participate, which is why the corresponding questionnaires (*n* = 14) were not considered within the analysis. Thus, 573 questionnaires were included in the evaluation (Table 2). Virtual encounters using the temi robot were used in 134 cases; in 167 cases, an alternative contact option (call, window visits, tablet) was used; and in 272 cases, no contact with relatives or friends took place. The number of virtual encounters using temi ranged from 1 to 15.

**Figure 2:**
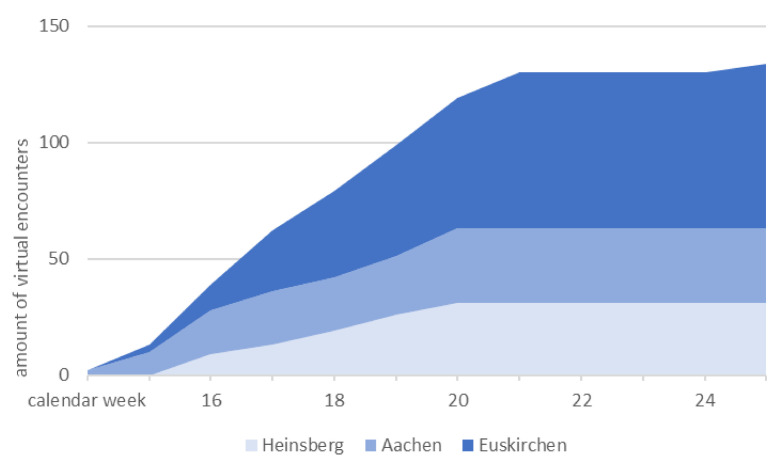
The cumulative number of virtual encounters increased rapidly during a “training period”, reaching a plateau after 6 to 7 weeks.

After an initial training period, the number of virtual encounters rapidly increased in all study sites (Figure 2).

**Figure 3:**
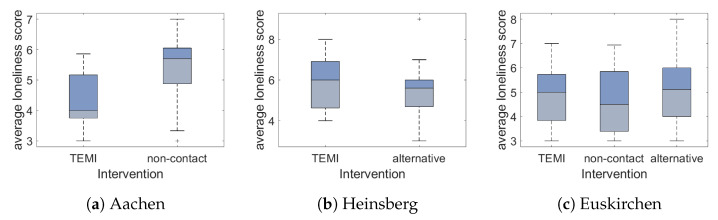
Average loneliness score. (**a**) A positive effect regarding loneliness was shown for the hospital study site. Here, the loneliness score was significantly decreased when virtual encounters took place compared to the control group (non-contact) (*p* = 0.01). Both in (**b**,**c**) there was no significant difference between the average loneliness score of the different intervention types.

Since the loneliness questionnaire used in this study has a point scale from 3 points (not lonely at all) to 9 points (totally lonely), a score of 6 points displays a neutral perception of loneliness. For each patient, the average score was determined for all questionnaires. Here, the median loneliness score was 5.3 (ranging from 3 to 9). In this study, 21% of our participants scored higher than 6. Within the longitudinal data, no significant changes of the loneliness scores were found. Therefore, the average loneliness score per volunteer and intervention over time was calculated. In the hospital group, the loneliness score was significantly lower among patients who used virtual encounters to meet friends and family virtually compared to among the non-contact group (F(1,25) = 7.783, *p* = 0.01, Figure 3a). For the nursing home in Euskirchen and the nursing home in Heinsberg, virtual encounters did not have any relevant effect regarding the loneliness score compared to the other study groups (Figure 3b,c).

The feedback from residents and patients using virtual encounters was positive. Depending on the allocation of roles, the use of virtual encounters with the help of temi had many advantages (Table 3).

## 4. Discussion

Social distance does not necessarily mean social isolation. Therefore, in this study, we wanted to make it easier for nursing home residents and hospital patients to contact their relatives by using a temi robot. Within the study period, 134 virtual encounters by means of temi were achieved. Furthermore, advantages of temi use could be determined for the different roles (temi users, relatives, nursing staff, and facility). Within the hospital, a lower perceived loneliness was also measured in the virtual encounter group compared to in the non-contact group by using the loneliness score.

Already in the first weeks of using temi to initiate video telephony between residents and their relatives, a steady, strong increase in usage was observed (Figure 1). Regarding the curve flattening from calendar week 20 (CW 20) onwards, the general ban on visits in Germany was lifted on 10 May 2020 (CW 20). This shows that video chats and temi robots could not generally replace personal contacts, although temi robots could be an additional option when personal contacts were not possible. Within the study, virtual encounters using temi were used up to 15 times per resident; feedback indicates that the residents found high usage of temi to be pleasant.

However, the general possibility to contact family members was also very positively received by the users. By avoiding physical contact, a possible infection can be avoided. This is particularly helpful for the elderly residents regarding the probability of a severe course in the case of an infection [22]. Compared to a tablet on which a video call could be initiated, the humanoid appearance and physical “visit” of the robot were also seen as very positive by the users. The same was true for anyone who saw the robot driving through the facility or where the robot was used as an entertainment object. In a Japanese study, a similar effect can be seen: with the help of the humanoid robot “Pepper” (SoftBank Robotics Group Corp., Paris, France), training units of the upper limb were offered to seniors, and their vital parameters were recorded. The test participants reacted to Pepper in a similar way to what would be expected with a human trainer [23]. Although robotic aid systems are commonly known in other countries such as Japan [24], in general usage is not yet necessarily given in practice [20]. With temi helping in nursing homes, we want to further promote digital innovation in medical institutions, even ones for elderly care. The self-sufficiency that residents gain with temi or other support robots also has a positive effect on nursing home residents, as the review by Sapci et al. shows [25]. Furthermore, regular contact using temi might also be helpful to alleviate the anxiety and worries of younger generations about their parents amid social distancing [26]. Direct contact with the nursing staff was also positively received by the relatives within the study. However, even after the ban on visitors vanishes, temi could be a tremendous improvement for families who live far away from their loved ones in nursing homes and are not able to see such family frequently. Health or financial hurdles that prevent regular visits to nursing homes can also be overcome by using video telephony, for example using temi, the respondents stated. Not only the users and their relatives but also the nursing staff were convinced of the benefits of the virtual encounters using temi. Compared to a tablet, which can also initiate video calls, temi offers the benefit of its contactless character based on autonomous driving and voice control. This reduces the workload of caregivers, as they no longer must perform certain transports or disinfection and thus have more time for the actual caregiving activity [27]. Even if the temi was operated via touch input, it could be disinfected without any problems, which is of course an important criterion in such a setting, especially during the COVID-19 pandemic.

According to the nursing staff, temi quickly became a “member” of the community. Although people need other people to be happy [28], objects are humanized when one is lonely, which is called anthropomorphism [29]. This can also be applied to the temi, which—due to the visitation bans increased loneliness—was perceived humanly and therefore accepted as part of the community. In addition, the possibly anthropomorphized human-robot communication is reinforced by the temi, since the temi also offers the possibility to get in contact with real humans. With respect to animals, it was found that animals are anthropomorphized with different degrees of ease [30] and a big factor is how cute they are perceived [31]. This, even though of course as robots and not animals, can hypothetically be applied to the temi, whose design and size also have aspects of cuteness. Thus, humanoid robots facilitate social integration and the desire for social contact, which, in the case of temi, again reinforces this need satisfaction through communication with family and friends [32]. Nevertheless, it is not only the appearance, but also the functionality—in a study, only externally no preference of the anthropomorphic over the functionally designed robot was drawn, if the functionality is not clear [33]. The function mode and the interaction with the robot accompanying with it lets the sympathy rise and thus anthropomorphism [34]. A further influence on anthropomorphism is the not understanding of the function mode (on seeing human), which can be released here naturally—due to the older, tendency technology-inexperienced generation. Just the anthropomorphic characteristics support the faster learning [30] and contribute so to the better handling of the application.

Within our study, positive feedback was also received from the facilities where the temi was set. According to the telephone interview, the use of digitization products thus increases the attractiveness of temi to an employer within a labor shortage. At the same time, the institution also benefits from the additional functionalities of the temi (e.g., playing individual music requests in common rooms or acting as a part of the community).

Even though the loneliness score was on average below the threshold of perceived loneliness, 21% of the volunteers scored higher than 6. At the same time, both extremes of the score were also scored by the volunteers. Thus, the maximum possible score of 9 was also achieved, which represents strong loneliness.

Although in fully residential facilities residents can build up a social network, elders are torn away from their familiar environment for a hospital stay. Therefore, a stay in a hospital is associated not only with anxiety but also sometimes with loneliness, particularly for older people. During the COVID-19 pandemic, visits to hospitals and even nursing homes had to be significantly restricted or temporarily prevented due to the associated risk of infection. Thus, in addition to visits from relatives, social contacts must be reestablished in the hospital, which is hardly possible within the time of the stay. Therefore, loneliness can especially increase due to a ban on visitors. Especially in these situations, the support of social contacts using virtual visits by family members and friends provided by a temi robot can be very helpful. The offer was very positively received and used by all parties. Therefore, this study shows that while patients are being treated in a hospital, a temi robot can counteract their loneliness significantly.

There are some limitations to be addressed. First, the sample size is low, which is why further studies are needed. In addition, a longer evaluation time would have enabled studying the long-term influence of a temi intervention. Whether long-term effects exist at all would also have to be investigated in further studies. Our intention besides scientific investigation was to fight social isolation during the visitor ban amid the COVID-19 pandemic. Another drawback is the irregular contact using temi between the volunteers and their relatives. A fixed schedule would have added value to the results here, but this is only partially feasible in practice.

Moreover, as an extension of the current application, residents could meet their family doctor or therapists via temi. This would not only reduce the risk of infection by medical staff but also increase appointment slots for the doctor due to decreased travel time. This would make it possible to avoid contact with therapists in times of a pandemic, which can pose an additional risk for residents and patients, without having to dispense with “talking” therapy (e.g., speech therapists, neurologists, psychotherapists). Additionally, virtual home visits by a general practitioner could be carried out by using special software (TeleDoc temi, Docs in Clouds TeleCare GmbH, Aachen, Germany).

## 5. Conclusions

The current work demonstrates that video telephony via an autonomously driving humanoid-resembling robot is a convenient chance to maintain the social network of nursing home residents or patients in clinics in times of a pandemic but also beyond, thus combating social isolation. It is precisely the older population group that is fundamentally affected by loneliness, which is why a visiting ban precisely affects these people and can lead to social isolation and loneliness. To counteract this, temi was used consistently and successfully within the study from the beginning. The advantages of using a temi robot extend to all user groups, including the elderly, relatives, and nursing staff of facilities, and regarding the consistently positive feedback, temi was soon considered a member of the group and brought joy to the elderly, even beyond its use for video telephony.

Temi can significantly reduce the social isolation of hospital patients. By comparison to residents of nursing homes, such patients have a shorter treatment time, and therefore there is no formation of a fixed social network in hospital institutions. Also, in nursing homes, temi can facilitate contact between residents and their relatives. Even if social isolation is a problem within the pandemic, it will continue to exist afterwards, which is why novel approaches as presented in this paper need to be further developed. 

## Figures and Tables

**Table 1:** Three-item loneliness score by Hughes et al. [17].

Question	Hardly Ever	Some of the Time	Often
How often do you feel that you lack companionship?	1	2	3
How often do you feel left out?	1	2	3
How often do you feel isolated from others?	1	2	3

**Table 2:** Overview of the number of completed questionnaires and the demographic data of participants of the study sites.

		Demographic				Study Groups			
		Age	Gender		Total	Intervention			Total
			Male	Female		TEMI	Non-Contact	Alternative	
	Heinsberg	83.6	8	32	40	31	0	135	166
Study sites	Aachen	80.4	8	11	19	32	77	0	109
	Euskirchen	82.5	3	8	11	71	195	32	298
	stotal	82.6	19	51	70	134	272	167	573

**Table 3:** Frequently expressed benefits related to virtual visits mediated by a robot.

Role	Benefits and Other Expressed Issues
Users	Capability to contact friends and family members frequently
	No risk of infection
	Embodiment of the visitor by the humanoid-resembling robot
	Maintaining social contacts
	Entertainment aspects of the robot (autonomous driving, speaking, etc.)
	Exciting event preventing boredom
	Voice control offers good operating comfort/high usability
	Possibility to show objects or their rooms during video telephony
Relatives	Continue to be in contact, despite the ban on visits
	Direct contact with nursing staff possible
	Feeling informed and can get a picture of the current state of health
	Fulfilment of the “duty” with simultaneous time saving
	No risk of infection
	No travel expenses (both financially and in terms of health)
Nursing staff	Direct contact with relatives
	Easier to handle than a tablet
	Temi can drive autonomously into quarantine rooms
	Temi can be left alone with the elderly: no supervision necessary.
	Temi surface able to be disinfected
	Entertainment capabilities (e.g., by providing YouTube content or music)
Facility	Increasing the attractiveness of employers (use of digitization products)
	Temi used in everyday life (temi as a part of the community)

## Data Availability

Data sharing not applicable.
